# Seizure Prevalence and Its Related Factors in Tramadol Intoxication; a Brief Report

**Published:** 2019-05-07

**Authors:** Asrin Babahajian, Payam Khomand, Farhad Manouchehri, Roozbeh Fakhimi, Behrooz Ahsan, Mohiadin Amjadian, Vahid Yousefinejad

**Affiliations:** 1 Liver and Digestive Research Center, Kurdistan University of Medical Sciences, Sanandaj, Iran.; 2 Department of Neurology, Faculty of Medicine, Kurdistan University of Medical Sciences, Sanandaj, Iran.; 3 School of Medicine, Kurdistan University of Medical Sciences, Sanandaj, Iran.; 4 Basic Science Department, Kurdistan University of Medical Sciences, Sanandaj, Iran.

**Keywords:** Tramadol, seizure, toxicity, emergency service, hospital, risk factor

## Abstract

**Introduction:**

Seizure is known to be a serious complication of tramadol consumption even in its therapeutic doses. The aim of this study was to determine the prevalence of seizure and its related factors in tramadol intoxicated patients referred to emergency department (ED).

**Methods:**

In this cross-sectional study, all individuals, admitted to ED following tramadol intoxication were divided into two groups based on the presence or absence of seizures. Demographic data as well as clinical, electroencephalogram and imaging findings were compared between the two groups using SPSS software version 22.

**Results:**

167 patients with the median age of 23 (13-45) years were studied (85% male). Seizure was seen in 97 (58.0%) cases. Risk of seizure had increased 3.7 times in patients with a history of seizure (OR: 3.71 Cl 95%: 1.17 - 11.76). Tramadol dose was significantly higher in patients who had seizure more than once (Median: 2800 IQR: 1800-4000), compared to those who had one seizure episode (Median: 850 IQR: 1800-400) (p <0.0001).

**Conclusion:**

Based on the findings of this study, history of seizure increased the risk of seizure in patients taking tramadol, and the increase in dose correlated with a significant increase in seizure frequency.

## Introduction:

Tramadol is a synthetic opioid drug with central effects, which is administered to relieve mild to severe pain. Tramadol is an analogue of 4-phenylpiperidine codeine. It exerts its therapeutic effects by influencing opioid receptors (μ), noradrenergic system, serotonergic system and GABAergic system, and also by inhibiting reuptake of norepinephrine in the central nervous system (1). Tramadol completely crosses the brain blood barrier. Its maximum plasma concentration is seen 90 minutes after oral administration. Its half-life is 5 to 6 hours. Its excretion is more renal and its therapeutic concentration in the blood is about 100-300 ng/ml (2). Tramadol can be given orally, subcutaneously, intravenously or suppository, but it has maximal absorption in oral administration. Therapeutic dose is 50 mg in oral, 50-100mg in injection and 100mg in anal administration. Also, the maximum daily dose should not exceed 400mg (3). Intoxication with tramadol may cause neurological complications such as seizure, respiratory arrest (apnea), and coma. The tramadol-induced seizure is often non-dose dependent and self-limited with generalized tonic-clonic feature (2). The neurotoxicity of tramadol occurs predominantly within the first 24 hours after ingestion and 84.6% of seizures occur in the first 6 hours after ingestion (4). The risk of seizure increases with concomitant use of tramadol with serotonin reuptake inhibitors (SSRIs), tricyclic antidepressants (TCAs), and monoamine oxidase MAO Inhibitors (5). 

Some studies have shown that tramadol could cause seizures in people with a history of epilepsy and in healthy people even at recommended doses, but most cases were observed among young people. Opiate dependency, drug abuse and their complications were common in all age groups of both males and females in Iran (6, 7). In recent years in Iran, excessive intake and intoxication with tramadol has been one of the most common causes of admissions in emergency wards (4, 8-10). An increase in seizure frequencies because of tramadol intoxication was also reported. It was reported that 15 to 35% of patients with tramadol intoxication experienced seizures (10, 11). Because of the increase in the number of tramadol users in recent years and, consequently, the increase of seizure associated with it, the prevalence of seizure and its related factors were evaluated in tramadol intoxicated patients in this study.

## Methods:

***Study design and setting***

This cross-sectional study was done in the emergency department of Tohid Hospital, Sanandaj, Iran, from March 2016 to August 2018. Tramadol intoxicated patients were divided into two groups based on the presence or absence of seizures, then demographic data as well as clinical, electroencephalogram and imaging findings were compared between the two groups. The protocol of the study was approved by the Ethics Committee of Kurdistan University of Medical Sciences (NOMUK1394/318). All participants gave written informed consent to participate in the study and the principles of confidentiality of data were respected by the researchers according to the Helsinki Declaration.

***Participants***

The study population consisted of all patients who had signs or symptoms of poisoning due to the use of tramadol (oral or injectable). Seizures with metabolic causes such as hypocalcemia or hypoglycemia were excluded from the study. Patients were selected and entered via consecutive sampling method.

***Data collection***

Demographic data, clinical findings, dosage and duration of tramadol intake, history of seizure, time interval between taking tramadol and manifestation of seizure, type of seizure, concomitant use of other drugs, as well as the frequency of seizures were collected using a researcher-made questionnaire by two general physicians. Then, the patients were divided into two groups based on the presence or absence of seizure. Computed tomography (CT) scan was done for all patients at the time of admission.

***Statistical analysis***

Minimum sample size required for the present study considering 95% confidence interval, 8% error, and 46.2% the prevalence of seizure following tramadol use (4), was calculated to be 150 cases. The results of the study were analyzed using SPSS V.22 software. Qualitative data were reported as frequency and percentages, and quantitative data were reported as median and interquartile range (IQR) due to abnormal distribution of data. Based on the incidence of seizure, patients were divided into two groups of with seizure and without seizure, then demographic characteristics and factors affecting seizure were compared in the two groups. To assess the relationship between quantitative and qualitative variables with seizure, Mann-Whitney U and Chi-square tests were used. Then, in order to determine the independent factors affecting seizure, variables with a p value** ˂**0.1 in the univariate analysis were entered into a multivariate logistic regression model and the data were reported as odds ratio (OR) with 95% confidence interval (CI). Spearman test was used to assess the correlation between tramadol intake dose and frequency of seizures. P˂0.05 was considered as significance level.

## Results:

167 patients with the median age of 23 (13-45) years were studied (85% male). All patients took oral tramadol and the median dose of tramadol was 1200 mg (100 to 12,000 mg). Seizure occurred in 97 (58.0%) patients. The frequency of seizure was one time in 80.4% and the type of seizure was a tonic-clonic type in 93.8% of cases. The median time interval between tramadol intake and seizure was 2 (1-12) hours.

There was not any significant relationship between seizure occurrence and age (p = 0.39) and gender (p = 0.28). The incidence of seizure was significantly higher in patients with a history of seizure (p = 0.03). Comparison of other risk factors between the two groups did not show any significant difference (Table 1).

In addition, the tramadol dose was significantly higher in patients who had seizures more than once (Median: 2800 IQR: 1800-4000), compared to those who had seizures only once (Median: 850 IQR: 1800-400) (r = 0.41; p <0.0001). The results of multivariate regression analysis showed that the history of seizure increased the risk of seizure in these patients by about four times (OR = 3.71; 95% CI: 1.17 to 11.76; p = 0.03) (Table 2).

## Discussion:

The findings of this study showed that 58.1% of patients with tramadol intoxication experienced seizures. The incidence of seizure was significantly higher in those with a history of seizure, and the dose of tramadol was significantly higher in patients who had experienced seizure more than once.

**Table 1:** Baseline characteristics of patients based of presence or absence of seizure

**variables**	**Seizure**		**p**
	**Without (n=97)**	**With (n=70)**	
**Age (year)**			
Median (percentile)	23.0 ( 21.0 -29.0 )	22.0 ( 19.0 – 30.0 )	0.39
**Gender **			
Female	12 (12.4)	13 (18.6)	0.28
Male	85 (87.6)	57 (81.4)	
**Tramadol intake (** **mg** **)**			
Median (percentile)	1000 (500– 2000)	1600 (800 -2850)	0.06
**Time to hospital admission (hour)**			
Median (percentile)	3.5 (2.0 – 5.0)	3.0 (2.0 -7.0)	0.35
**History of seizure**			
Yes	17 (17.7)	4 (5.7)	0.03
No	79 (82.3)	66 (94.3)	
**Concomitant use of opioids**			
Yes	5 (5.2)	9 (12.9)	0.09
No	92 (94.8)	61 (87.1)	
**Concomitant use of other drugs**			
Yes	6 (6.2)	4 (5.7)	0.99
No	91 (93.8)	66 (94.3)	
**Episodes of seizure **			
Single seizure	78 (80.4)	-	NA
Multiple seizures	19 (19.6)	-	

Data are presented as median (percentile 25-75) or frequency (%). NA: not applicable.

**Table 2:** Independent risk factors of seizure following tramadol intoxication

**Variables**	**Odds ratio**	**95% CI**	**p**
History of seizure	3.71	1.17 – 11.76	0.03
Concomitant use of opioids	0.35	0.11 – 1.13	0.08

Tramadol intoxication rate showed a significant increase in Iran like any other part of the world (12, 13), and in some studies in Iran, the incidence of seizure was reported to be high in patients with tramadol intoxication. Although excessive intake of tramadol was not life threatening, seizures sometimes increased mortality in these individuals (14-16). Talaie et al., reported the incidence of seizure to be 46.2% (4). In another study, 48% of the 144 patients with tramadol intoxication experienced seizures (17). 

The precise mechanism of tramadol’s effect on seizure induction has not yet been determined. Research showed that at high doses, tramadol had inhibitory effects on gamma aminobutyric acid (GABA) receptors (18, 19), and in animal models, inhibition of GABA receptors increased seizure severity (20). In addition, seizure was known to be a major side effect of tramadol which might occur with the use of both therapeutic and toxic doses (4, 21-26). Also, the direct effect of tramadol on seizure has not been fully proven.

In the present study, the median dose of tramadol was 1,000 mg (ranging from 100 to 1200 mg) in patients with seizure, which showed that none of the seizures was induced by the therapeutic doses, but when compared with the doses used in patients without seizure, there was no significant difference (P=0.06). This data showed that incidence of seizure in the patients was not dose-dependent. However, with an increase in the dose, the frequency of seizures increased.

The dose of tramadol intake in patients who had more than one seizure was three times more than those with only one seizure, which showed a moderate correlation between drug dose and frequency of seizures. In the study by Shadnia et al., in patients who had seizures more than once, tramadol intake dose was significantly higher compared to those who had seizure only once, which is consistent with the results of this study (24). However, in our study the correlation between the intake dose and the frequency of seizure was poor. Considering that in both studies the tramadol dose assessment was done based on asking the patient or his relatives, confirmation of the relationship between the dose and the frequency of seizure requires more detailed studies.

Also, the results of this study showed that the risk of seizure had increased in patients with a history of seizure. Based on this finding, it is recommended to take history of seizure in patients who might need tramadol administration, especially for long-term use.

The limitation of this study was that the dose of the drug taken by each patient was determined based on his/her self-declaration, which is not very accurate and reliable.

## Conclusion:

Based on the findings of this study, a history of seizure increased the risk of seizure in patients taking tramadol, and an increase in the dose correlated with a significant increase in seizure frequency. It is recommended to take an accurate history of seizure in patients who might need tramadol administration, especially for long-term use.
